# Nontuberculous mycobacterial pulmonary disease diagnosed by two methods: a prospective cohort study

**DOI:** 10.1186/s12879-019-4078-0

**Published:** 2019-05-24

**Authors:** Hyung-Jun Kim, Jong Hyuk Lee, Soon Ho Yoon, Sung A. Kim, Myoung Sil Kim, Sun Mi Choi, Jinwoo Lee, Chang-Hoon Lee, Sung Koo Han, Jae-Joon Yim

**Affiliations:** 1 Division of Pulmonary and Critical Care Medicine, Department of Internal Medicine, Seoul National University College of Medicine, Seoul National University Hospital, 101 Daehak-Ro Jongno-Gu, 03080 Seoul, Republic of Korea; 2 Department of Radiology, Seoul National University College of Medicine, Seoul National University Hospital, Seoul, Republic of Korea

**Keywords:** Clinical medicine, Cohort studies, Diagnosis, Nontuberculous mycobacteria, Progression

## Abstract

**Background:**

Microbiological criteria for diagnosing nontuberculous mycobacterial pulmonary disease (NTM-PD) include positive culture results from at least two separately expectorated sputum specimens or one bronchial washing or lavage. However, the clinical similarities and differences between patients diagnosed by these two methods remain unclear. We compared clinical features and prognoses of patients with NTM-PD diagnosed from both specimen types.

**Methods:**

We analysed data from patients who participated in the Seoul National University Hospital NTM-PD cohort (ClinicalTrials.gov identifier: NCT01616745). Baseline demographics, symptoms, radiographic findings, disease progression, and treatment responses were summarized and compared between patients diagnosed from sputum specimens and patients diagnosed from bronchoscopic specimens.

**Results:**

Three hundred forty-seven patients were included in the analyses. Of these, 279 (80.4%) were diagnosed from two separately expectorated sputum specimens, and 68 (19.6%) were diagnosed from bronchoscopic specimens. Patients diagnosed from sputum specimens had more frequent and severe cough, sputum, postnasal drip, and high St. George’s Respiratory Questionnaire scores. However, the extent and severity of the radiographic lesions, disease progression, and treatment responses were similar for both groups. Further analysis based on the following three groups (sputum culture positive, sputum culture negative/bronchoscopy, and scanty sputum/bronchoscopy groups) suggested that the scanty sputum/bronchoscopy group appeared to have the worst prognosis in terms of both time to progression and time to culture conversion.

**Conclusions:**

Although some symptoms and quality of life were worse in patients with NTM-PD diagnosed from sputum specimens, their prognoses were similar to those of patients diagnosed by bronchoscopic specimen. We recommend bronchoscopic sampling for patients in whom NTM-PD is suspected clinically or radiographically, especially those who have no or scanty sputum.

**Electronic supplementary material:**

The online version of this article (10.1186/s12879-019-4078-0) contains supplementary material, which is available to authorized users.

## Background

The incidence and prevalence of nontuberculous mycobacterial (NTM) pulmonary disease (PD) is increasing worldwide [1–8]. In South Korea, the prevalence of NTM infections has also increased rapidly: 9.4/100,000 persons in 2009 to 36.1/100,000 persons in 2016 [9]. Increases in NTM-PD lead to more hospitalizations, higher costs, and increased mortality [10].

Diagnosing NTM-PD relies on combining clinical and microbiological criteria [11, 12]. Clinical criteria include pulmonary symptoms and nodular or cavitary opacities on chest imaging, with appropriate exclusion of other diagnoses. To fulfil the microbiological criteria, positive cultures from at least two separately expectorated sputum specimens or one bronchial washing or lavage specimen are required.

However, it remains unclear whether the clinical features differ between NTM patients diagnosed by the two methods [11]. Diagnosis using sputum may imply the presence of productive sputum, which may represent more severe form of NTM-PD. Furthermore, the current guidelines do not propose any clinical evidence about the diagnostic criteria of NTM-PD [11, 12]. This study compared clinical characteristics and prognoses between patients diagnosed from either two sputum specimens or from one bronchoscopic specimen.

## Methods

### Study participants and design

Patients aged ≥20 years with NTM-PD who participated in the ongoing prospective cohort, Seoul National University Hospital NTM cohort (ClinicalTrials.gov identifier: NCT01616745), which commenced on 1 July 2011, were included in this analysis [13–16]. NTM-PD was diagnosed in accordance with the criteria proposed by the American Thoracic Society/Infectious Diseases Society of America (ATS/IDSA) and British Thoracic Society (BTS) guidelines [11, 12]. Bronchoscopic sampling was performed in patients who could not expectorate or in patients with suspected NTM-PD but negative mycobacterial culture. Patients who withdrew consent, were previously treated for NTM-PD, or had poor compliance during treatment (delaying visits for > 1 month) were excluded. All patients provided written informed consent before registering. Baseline data were retrieved on 11 April 2017, and the follow-up data on disease progression and culture conversion were retrieved on 26 January 2018.

### Baseline evaluations

Baseline demographics were recorded at the time of enrolment. Cough and sputum symptoms were divided into four categories: none, sometimes, often, and almost every day for coughing; none, ≤3 days per week, ≥4 days per week, and almost every day for sputum. Dyspnoea scales were divided into five categories (0–4) per the modified Medical Research Council dyspnoea scale [17]. Other complaints such as haemoptysis, postnasal drip, fever, and weight loss were also recorded. Anxiety and depression were evaluated using the Hospital Anxiety and Depression Scale [18]. Overall respiratory health and quality of life were assessed utilizing St. George’s Respiratory Questionnaire [19].

Baseline serum laboratory tests were performed, and pulmonary function was tested for forced expiratory volume in 1 second (FEV_1_), forced vital capacity (FVC), FEV_1_/FVC ratio, and diffusing capacity for carbon monoxide. Computed tomography (CT) scans of the chest were obtained upon diagnosis. Radiographic subtype was classified as nodular bronchiectatic form or upper lobe cavitary form. When the subtype was unclassifiable, it was allocated into either subtype based on the dominant feature for further analysis in this study. In addition, radiographic NTM-PD severity was assessed by two board-certified radiologists, who were blinded to the patients’ clinical data, using a previously introduced scoring system [20]. The extents of bronchiectasis (maximum score of 9), cellular bronchiolitis (maximum score of 6), cavities (maximum score of 9), nodules (maximum score of 3), and consolidation (maximum score of 3) were considered in calculating the total score (maximum score of 30).

### Follow-up evaluations

Participants were followed every 3 to 6 months. On each visit, participants were examined by investigators who obtained sputum specimens for acid-fast bacilli smears and mycobacterial cultures and took simple chest radiographs. CT scans of the chest were repeated within one year after enrolment, then every two years, and radiographic severity was reassessed upon disease progression using the scoring system described above. Once treatment was initiated, participants were followed every 4–8 weeks. On each visit, participants were examined, sputum specimens were obtained and simple chest radiographs were taken.

### Microbiological assessment

Acid-fast bacilli smears and mycobacterial cultures were performed following the recent guidelines [11]. All cultures were grown in both solid Ogawa media (Shinyang Diagnostics, Seoul, South Korea) and the BACTEC MGIT 960 system (Becton-Dickinson and Co., Sparks, MD, US). NTM species were identified by sequencing of the 16S ribosomal RNA gene using the algorithm specified in the Clinical and Laboratory Standards Institute guidelines [13], as well as sequencing of the rpoB gene [21]. rpoB gene sequencing and patterns of clarithromycin resistance were checked to differentiate between *M. abscessus* subspecies *abscessus* and *M. abscessus* subspecies *massiliense* [22]. *M. abscessus* subspecies taxonomy was in accordance with recent suggestions [23]. Patients were considered to have mixed NTM species infections if an NTM species other than the original species was isolated at least twice during the follow-up period.

### Definitions

Patients were divided into two groups per the diagnostic method upon enrolment: either isolation of NTM from ≥2 sputum specimens (sputum group) or isolation of NTM from a bronchoscopic specimen (bronchoscopy group). When NTM were isolated from both sputum and bronchoscopic specimens, patients were classified by the test that yielded NTM sooner. For the sputum group, the date of the second positive sputum culture was considered the date of diagnosis. For the bronchoscopy group, the date of the bronchoscopy that yielded a positive NTM culture was considered the date of diagnosis.

Disease progression was defined as physician recommendation for treatment initiation based on clinical and radiographic deterioration, as used previously by us and other researchers [16, 24, 25]. Decision to initiate treatment was made by the on-duty physicians based on clinical (e.g., new-onset haemoptysis) or radiographic deterioration (e.g., cavity formation) for each patient. Four physicians (CSM, LJ, LCH, and YJJ) managed the patients and discussed the need to initiate treatment.

NTM-PD was treated as per recent guidelines [11, 12]. Clarithromycin-sensitive *Mycobacterium avium* complex (MAC)-PD was treated with a macrolide, ethambutol, and rifampicin using a thrice weekly or daily schedule, and an injectable aminoglycoside was considered in patients with a severe disease extent. *M. abscessus*-PD was initially treated with a combination of azithromycin, aminoglycoside, and imipenem (or cefoxitin) for the first 3 weeks, and aminoglycoside administration (3 to 5 times per week) was continued for at least 3 more months with azithromycin during the continuation phase. Clofazimine has been used for both MAC-PD and *M. abscessus*-PD patients who were refractory to treatment or resistant to clarithromycin. When treating patients with mixed NTM infections, we targeted the most frequently isolated NTM species.

Culture conversion was defined as at least three consecutive negative results from sputum mycobacterial cultures after initiating treatment and adopted as a parameter for treatment response [26]. The collection day of the first sputum with a negative culture was regarded as the day of culture conversion [26, 27].

### Statistical analysis

Categorical variables were summarized as numbers with percentages and compared using a chi-square test or Fisher’s exact test. Continuous variables are presented as the median with interquartile range (IQR) and analysed using the Mann-Whitney U test. The Wilcoxon signed-rank test was also used to evaluate changes in NTM-PD radiographic severity scores. To estimate the probability of disease progression and culture conversion after treatment, Kaplan-Meier survival analyses with a log-rank test were used. To estimate hazard ratios (HRs) with 95% confidence intervals (CIs) for each potential variable affecting the study outcomes, we utilized a Cox proportional hazards model. Covariates with *P*-values less than 0.05 were further included in a multivariate model to calculate the adjusted HR of each variable. All statistical analyses were performed using Stata, version 13.0 (Stata Corp., College Station, TX, US).

## Results

### Baseline characteristics

On the date of extraction of initial data (11 April 2017), 372 patients were enrolled in the cohort. Of these, patients who did not fulfil the diagnostic criteria for NTM-PD (*n* = 11), had withdrawn consent (*n* = 10), started treatment before diagnosis (*n* = 3), or had poor compliance (n = 1) were excluded. Finally, 347 patients with NTM-PD were included in the analysis. Of these, 279 (80.4%) were diagnosed from two consecutive sputum specimens obtained at a median interval of 3.2 months (IQR 0.3, 0.7), whereas 68 (19.6%) were diagnosed by examination of single bronchoscopic specimens. The reasons for undergoing bronchoscopic sampling were as follows: could not expectorate or had too little sputum (47 patients) or suspected NTM-PD but negative acid-fast staining or mycobacterial culture (21 patients) on ≥2 sputum specimens.

Sex and age were similarly distributed between both groups. Patients in the sputum group smoked more cigarettes (median 22.5 pack-years [IQR: 15, 33.8]) than did the bronchoscopy group (median 13.8 [IQR: 5, 20], *P* = 0.046). History of tuberculosis treatment was more common in the sputum group than in the bronchoscopy group (41.6% vs. 22.1%, *P* = 0.008). Nodular bronchiectatic form was the main radiographic subtype in both groups: 90.0 and 94.1% in the sputum and bronchoscopy groups, respectively (*P* = 0.288). NTM species were similarly distributed in both groups. MAC was the most common (*n* = 243), and *M. abscessus* the second most common (*n* = 63). *M. abscessus* consists of 32 strains of *M. abscessus* subsp. *abscessus* and 31 of *M. abscessus* subsp. *massiliense*. Twelve of 32 strains of *M. abscessus* subsp. *abscessus* (37.5%) and 26 strains of 31 *M. abscessus* subsp. *massiliense* (83.9%) were clarithromycin-susceptible. The rate of smear positivity did not differ according to the type of specimen: 16.9 and 22.1% were positive in the sputum and bronchoscopy group, respectively (*P* = 0.314) (Table 1).

**Table 1:** Baseline characteristics of 347 patients diagnosed with nontuberculous mycobacterial pulmonary disease using different diagnostic methods

Variables	Total patients *N* = 347	Sputum group *n* = 279	Bronchoscopy group *n* = 68	*P-*value
Sex, female	215 (62.0)	176 (63.1)	39 (57.4)	0.383
Age, years	66 (59, 74)	67 (59, 74)	66 (58, 74)	0.509
Smoking history				0.985
Never smoker	258 (74.4)	208 (74.6)	50 (73.5)	
Former smoker	79 (22.8)	63 (22.6)	16 (23.5)	
Current smoker	10 (2.9)	8 (2.9)	2 (2.9)	
Pack-years for smokers	20 (10, 30)	22.5 (15, 33.8)	13.8 (5, 20)	0.046
History of tuberculosis	131 (37.8)	116 (41.6)	15 (22.1)	0.008
Smear positivity of specimen on diagnosis	62 (17.9)	47 (16.9)	15 (22.1)	0.314
Underlying disease				
COPD	32 (9.2)	28 (10.0)	4 (5.9)	0.288
GERD	27 (7.8)	23 (8.3)	4 (5.9)	0.583
Cancer	22 (6.3)	18 (6.5)	4 (5.9)	0.863
Diabetes mellitus	22 (6.3)	16 (5.7)	6 (8.8)	0.349
Occupational status				0.583
Office work	53 (15.3)	42 (15.1)	11 (16.2)	
Housewife	53 (15.3)	41 (14.7)	12 (17.7)	
Professional practitioner	41 (11.8)	33 (11.8)	8 (11.8)	
Self-employment	38 (11.0)	32 (11.5)	6 (8.8)	
Unemployed	21 (6.1)	18 (6.5)	3 (4.4)	
Construction worker	14 (4.0)	13 (4.7)	1 (1.5)	
Service industry	14 (4.0)	12 (4.3)	2 (2.9)	
Radiographic subtype				0.288
Nodular bronchiectatic form	315 (90.8)	251 (90.0)	64 (94.1)	
Upper lobe cavitary form	32 (9.2)	28 (10.0)	4 (5.9)	
NTM species				0.184
*Mycobacterium avium*	129 (37.2)	97 (34.8)	32 (47.1)	
*Mycobacterium intracellulare*	110 (31.7)	91 (32.6)	19 (27.9)	
*Other MAC*	4 (1.2)	4 (1.4)	0 (0.0)	
*Mycobacterium abscessus subsp. abscessus*	32 (9.2)	25 (9.0)	7 (10.3)	
*Mycobacterium abscessus subsp. massiliense*	31 (8.9)	28 (10.0)	3 (4.4)	
*Mycobacterium kansasii*	7 (2.0)	6 (2.2)	1 (1.5)	
*Mycobacterium fortuitum*	3 (0.9)	3 (1.1)	0 (0.0)	
Others	13 (3.7)	10 (3.6)	3 (4.4)	
Mixed infection^a^	12 (3.5)	12 (4.3)	0 (0.0)	
Unknown^b^	6 (1.7)	3 (1.1)	3 (4.4)	

Values are presented as number (percentage) or median (interquartile range)

Abbreviations: *COPD* chronic obstructive pulmonary disease, *GERD* gastroesophageal reflux disease, *NTM* nontuberculous mycobacteria, *MAC Mycobacterium avium* complex

^a^ More than two NTM species isolated at least twice each during the follow-up period until the start of treatment. ^b^The results of species identification for NTM are missing

FEV_1_ was lower in the sputum group than in the bronchoscopy group, although this was not statistically significant (2.12 L vs.2.33 L, *P* = 0.06). Other laboratory test details revealed no significant differences between the two groups [see Additional file 1: Table S1].

### Differences in subjective symptoms upon NTM-PD diagnosis

Coughing and sputum were more severe among patients in the sputum group, in which, 76 patients (27.2%) had coughing “almost every day”, compared with 11 (16.2%) in the bronchoscopy group (*P* = 0.002). Likewise, 140 patients (50.2%) in the sputum group had sputum “almost every day”, compared with only 14 (20.6%) in the bronchoscopy group (*P* < 0.001). Patients in the sputum group had more severe respiratory symptoms and worse quality of life overall than those in the bronchoscopy group, showing higher St. George’s Respiratory Questionnaire scores (median 17.6 vs. 9.9, respectively, *P* < 0.001). The frequencies of other symptoms, including dyspnoea, haemoptysis, fever, weight loss, anxiety, and depression were similar between the groups (Table 2).

**Table 2:** Differences in assessed symptoms of nontuberculous mycobacterial pulmonary disease between patients diagnosed using different methods upon enrolment

Variables	Total patients *N* = 347	Sputum group *n* = 279	Bronchoscopy group *n* = 68	*P-*value
Cough				0.002
None	129 (37.2)	90 (32.3)	39 (57.4)	
Sometimes	77 (22.2)	67 (24.0)	10 (14.7)	
Often	54 (15.6)	46 (16.5)	8 (11.8)	
Almost everyday	87 (25.1)	76 (27.2)	11 (16.2)	
Sputum				< 0.001
None	105 (30.3)	66 (23.7)	39 (57.4)	
≤ 3 days per week	53 (15.3)	43 (15.4)	10 (14.7)	
≥ 4 days per week	35 (10.1)	30 (10.8)	5 (7.4)	
Almost everyday	154 (44.4)	140 (50.2)	14 (20.6)	
mMRC dyspnoea scale				0.581
0	131 (37.9)	102 (36.7)	29 (42.7)	
1	197 (56.9)	159 (57.2)	38 (55.9)	
2	15 (4.3)	14 (5.0)	1 (1.5)	
3	2 (0.6)	2 (0.7)	0 (0.0)	
4	1 (0.3)	1 (0.4)	0 (0.0)	
Haemoptysis	63 (18.2)	50 (17.9)	13 (19.1)	0.864
Postnasal drip	92 (26.5)	84 (30.1)	8 (11.8)	0.007
SGRQ score	16.1 (8.1, 30.6)	17.6 (9.5, 32.4)	9.9 (4.9, 20.2)	< 0.001
Fever	39 (11.2)	32 (11.5)	7 (10.3)	0.850
Weight loss	30 (8.7)	25 (9.0)	5 (7.4)	0.800
Anxiety scale	5 (2, 7)	5 (2, 7)	5 (2, 7)	0.632
Depression scale	5 (2, 7)	5 (2, 8)	5 (3, 7)	0.716

Values are presented as number (percentage) or median (interquartile range)

Abbreviations: *mMRC* modified Medical Research Council, *SGRQ* St. George’s Respiratory Questionnaire

### Disease progression among patients with NTM-PD

Patients were followed for a median of 2.9 years (IQR: 1.6, 4.6) after their NTM-PD diagnosis: median 3.0 years (IQR:1.8, 4.8) for the sputum group vs. 2.1 years (IQR: 1.1, 3.7) for the bronchoscopy group. Among the 347 patients, 133 (38.3%) experienced disease progression. In the sputum group, disease progression manifested as clinical deterioration in 17 patients (15.3%), radiographic deterioration in 54 (48.7%), and both in 40 (36.0%). The ratios were similar in the bronchoscopy group, namely 4 (18.2%), 11 (50.0%), and 7 (31.8%) patients, respectively, in the above three categories (*P* = 0.907).

**Fig. 1:**
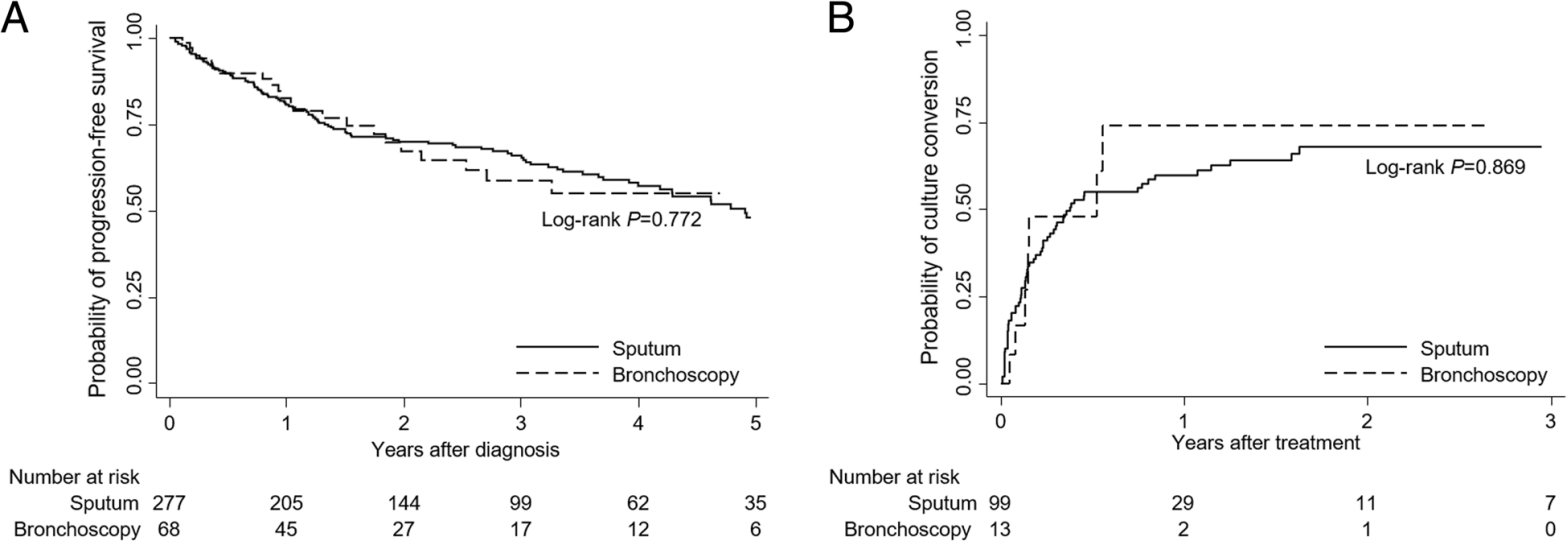
Comparison of disease progression and time to culture conversion between patients diagnosed from sputum specimens and patients diagnosed from bronchoscopic specimen. **a** Probability of progression-free survival. **b** Probability of culture conversion after treatment

Time to progression for NTM-PD was similar between the sputum and bronchoscopy groups (log-rank *P* = 0.772) (Fig. 1a). In the sputum group, one-year and two-year progression rates were 19.0 and 28.0%, while in the bronchoscopy group, they were 16.2 and 26.5%. Times to progression according to diagnostic methods were similar between MAC-PD (log-rank *P* = 0.987) and *M. abscessus*-PD (log-rank *P* = 0.455) patients. Younger age (HR 0.97; 95% CI 0.96–0.99 per year) and the upper lobe cavitary disease form (HR 3.16; 95% CI 1.78–5.62 compared with the nodular bronchiectatic form) were identified as independent factors for earlier disease progression. However, diagnostic method (sputum vs. bronchoscopy) was not a predictor of earlier disease progression; the HR of the bronchoscopy group was 0.82 with a 95% CI 0.48–1.38 compared with the sputum group (Table 3).

**Table 3:** Factors associated with disease progression of nontuberculous mycobacterial lung disease

Variables	Unadjusted HR (95% CI)	Adjusted HR (95% CI)
Age, by each year	0.97 (0.96–0.99) ^*^	0.97 (0.96–0.99) ^*^
Sex, female	1.38 (0.96–2.00)	–
Body mass index, by 1 kg/m^2^	0.89 (0.83–0.95) ^*^	0.94 (0.87–1.01)
Smoking		
Never smoker	Reference	–
Former smoker	0.72 (0.47–1.18)	–
Current smoker	1.82 (0.80–4.15)	–
Haemoglobin, by 1 g/dL	0.83 (0.73–0.95) ^*^	0.89 (0.75–1.06)
Platelet, by 1000/μL	1.00 (1.00–1.01) ^*^	1.00 (1.00–1.00)
Total protein, by 1 g/dL	1.88 (1.22–2.89) ^*^	1.57 (0.97–2.52)
Total bilirubin, by 1 mg/dL	0.40 (0.18–0.85) ^*^	0.42 (0.17–1.04)
SGRQ score, by each score	1.01 (1.00–1.02)	–
FEV_1_, by 1 L	1.00 (1.00–1.00)	–
FVC, by 1 L	1.00 (1.00–1.00)	–
DLCO, by 1%	0.99 (0.98–1.00) ^*^	0.99 (0.98–1.01)
Computed tomography type		
Nodular bronchiectatic form	Reference	Reference
Upper lobe cavitary form	3.69 (2.30–5.94) ^*^	3.16 (1.78–5.62) ^*^
Diagnostic method		
NTM culture on two sputum specimens	Reference	Reference
NTM culture on bronchoscopic specimen	0.93 (0.59–1.48)	0.82 (0.48–1.38)

^*^ Indicates statistical significance with *P*-value< 0.05

Abbreviations: *SGRQ* St. George’s Respiratory Questionnaire, *FEV*_*1*_ forced expiratory volume in 1 second, *FVC* forced vital capacity, *DLCO* diffusing capacity of the lungs for carbon monoxide

The baseline radiographic severity score showed a median of 8 (IQR: 6, 10), which was elevated to median 10 (IQR: 7, 14) with disease progression (*P* < 0.001 by paired *t*-test). These scores ranged from median 8 (IQR: 6, 10) to 10 (IQR: 7, 14) for the sputum group (*P* < 0.001) and from median 8 (IQR: 6, 11) to 10 (IQR: 7, 14) for the bronchoscopy group (*P* = 0.002).

### Treatment response among NTM patients in whom treatment was initiated

The 133 patients who experienced disease progression were further analysed. Of these, 131 patients began treatment, while two (1.5%) refused, and 78 (58.6%) remained on treatment at the time of data retrieval. The median treatment duration was 1.4 years (IQR: 0.7, 1.7) in all patients who initiated treatment, with medians of 1.5 years (IQR 0.8, 1.8) for the sputum group and 1.2 years (IQR 0.2, 1.4) for the bronchoscopy group.

Time to culture conversion was compared between the 13 patients in the bronchoscopy group whose subsequent sputum cultures yielded NTM and who therefore started treatment and the 109 patients in the sputum group who also started treatment. Culture conversion of sputum was achieved in 7 of 13 patients in the bronchoscopy group (53.9%) within a median of 1.8 months (IQR: 1.0, 6.4) and in 71 of 109 patients in the sputum group (65.1%) within a median of 1.8 months (IQR: 0.5, 4.3). Time to culture conversion did not differ significantly between the sputum and bronchoscopy groups (log-rank *P* = 0.869) (Fig. 1b). Time to culture conversion according to diagnostic method also did not differ between patients with MAC-PD (log-rank *P* = 0.440) and *M. abscessus*-PD (log-rank *P* = 0.267*).*

### Comparison of groups according to reasons for undergoing bronchoscopy

Because there were two different categories of reasons for undergoing bronchoscopy, namely negative sputum culture and scanty sputum, further analysis was performed based on the following three groups: sputum culture positive, sputum culture negative/bronchoscopy, and scanty sputum/bronchoscopy groups (Table 4).

**Table 4:** Characteristics of 347 patients diagnosed with nontuberculous mycobacterial pulmonary disease according to three groups of patients

Variables	Sputum culture positive *n* = 279	Sputum culture negative/Bronchoscopy *n* = 21	Scanty sputum/Bronchoscopy *n* = 47	*P*-value
Sex, female	176 (63.1)	10 (47.6)	29 (61.7)	0.371
Age, years	67 (59, 74)	64 (60, 76)	66 (57, 73)	0.593
Smoking history				0.919
Never smoker	209 (74.6)	15 (71.4)	35 (74.5)	
Former smoker	63 (22.6)	5 (23.8)	11 (23.4)	
Current smoker	9 (2.9)	1 (4.8)	1 (2.1)	
History of tuberculosis	116 (41.6)	6 (28.6)	9 (19.2)	0.034
Smear-positive upon diagnosis	47 (16.9)	2 (9.5)	13 (27.7)	0.311
Underlying disease				
COPD	28 (10.0)	3 (14.3)	1 (2.1)	0.092
GERD	23 (8.2)	1 (4.8)	3 (6.4)	0.712
Cancer	18 (6.5)	3 (14.3)	1 (2.1)	0.155
Diabetes mellitus	16 (5.7)	4 (19.1)	2 (4.3)	0.069
Radiographic subtype				0.475
Nodular bronchiectatic	251 (90.0)	19 (90.5)	45 (95.7)	
Upper lobe cavitary	29 (10.0)	2 (9.5)	2 (4.3)	
Cough				0.004
None	90 (32.3)	8 (38.1)	31 (66.0)	
Sometimes	67 (24.0)	5 (23.8)	5 (10.6)	
Often	46 (16.5)	3 (14.3)	5 (10.6)	
Almost everyday	76 (27.2)	5 (23.8)	6 (12.8)	
Sputum				< 0.001
None	66 (23.7)	13 (61.9)	26 (55.3)	
≤ 3 days per week	43 (15.4)	2 (9.5)	8 (17.0)	
≥ 4 days per week	30 (10.8)	1 (4.8)	4 (8.5)	
Almost everyday	140 (50.2)	5 (23.8)	9 (19.2)	
mMRC dyspnoea scale				0.806
0	102 (36.7)	7 (33.3)	22 (46.8)	
1	159 (57.2)	14 (66.7)	24 (51.1)	
2	14 (5.0)	0 (0.0)	1 (2.1)	
3	2 (0.7)	0 (0.0)	0 (0.0)	
4	1 (0.4)	0 (0.0)	0 (0.0)	
Haemoptysis	50 (17.9)	5 (23.8)	8 (17.0)	0.792
Postnasal drip	84 (30.1)	3 (14.3)	5 (10.6)	0.015
SGRQ score	17.6 (9.5, 32.4)	12.6 (6.9, 21.0)	9.2 (4.1, 17.5)	< 0.001
Fever	32 (11.5)	3 (14.3)	4 (8.5)	0.807
Weight loss	25 (9.0)	1 (4.8)	4 (8.5)	> 0.999
Anxiety scale	5 (2, 7)	4 (2, 7)	5 (2, 6)	0.872
Depression scale	5 (2, 8)	5 (3, 7)	5 (3, 7)	0.917

Values are presented as number (percentage) or median (interquartile range)

Abbreviations: *COPD* chronic obstructive pulmonary disease, *GERD* gastroesophageal reflux disease, *mMRC* modified medical research council, *SGRQ* St. George’s Respiratory Questionnaire

**Fig. 2:**
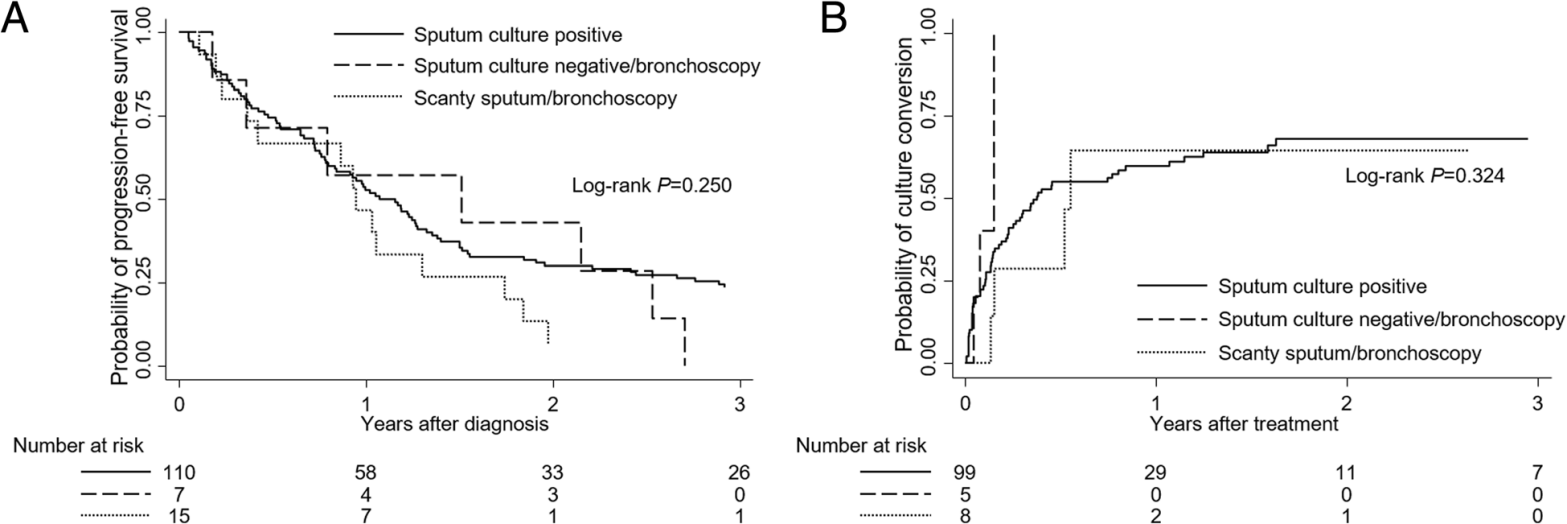
Comparison of disease progression and time to culture conversion according to three groups: sputum culture positive, sputum culture negative/bronchoscopy, and scanty sputum/bronchoscopy group. **a** Probability of progression-free survival. **b** Probability of culture conversion after treatment

Although time to progression did not differ significantly between these three groups (log-rank *P* = 0.250) (Fig. 2a) and nor did time to culture conversion after treatment (log-rank *P* = 0.324) (Fig. 2b), the prognosis appeared to be worst in the scanty sputum/bronchoscopy group.

## Discussion

Due to the ubiquitous nature of NTM species, multiple positive culture results from expectorated sputum should be analysed [28]. In addition, more than two MAC isolates were reported to be linked to development of new cavities or progression of infiltration [29]. Conversely, specimens from bronchoscopic washing or bronchoalveolar lavage taken from disease-specific sites may lower the contamination risk and increase the specificity of the culture result [30]. Therefore, guidelines recommend that single NTM isolates from bronchoscopic specimens satisfy microbiological criteria [11, 12]. BTS guidelines recommend CT-directed bronchial washing for NTM suspects who cannot expectorate sputum or whose sputum is consistently culture-negative [12].

In this prospective cohort study, clinical features and prognoses were compared for NTM-PD patients diagnosed from two different microbiological criteria (two sputum specimens or one bronchoscopic specimen). Although some symptoms and quality of life were worse in patients diagnosed from sputum, times to disease progression and culture conversion with treatment were similar between both groups.

The similar prognoses despite worse symptoms found in this study for patients diagnosed from sputum requires further explanation. Sputum group patients more frequently complained of symptoms related to sputum production or expectoration (e.g., cough, sputum, postnasal drip). However, other symptoms not directly associated with sputum yield (e.g., haemoptysis, fever, weight loss) were similar between both groups. Results of other tests, including pulmonary function and radiographic severity, were also similar. Finally, disease progression and culture conversion with treatment were similar between both groups. These findings suggest that the specimen collection method reflects the sputum accessibility, rather than the overall disease severity. Our results support the microbiologic criteria for NTM-PD suggested by the current ATS/IDSA and BTS guidelines [11, 12].

It is interesting to note the differences between the sputum culture negative/bronchoscopy and scanty sputum/bronchoscopy groups. Our analysis suggested that the scanty sputum/bronchoscopy group appeared to have the worst prognosis in terms of both time to progression and time to culture conversion; however, this difference was not statistically significant. This finding suggests that bronchoscopy should be performed more actively in patients with possible NTM-PD who are not producing sputum.

In our institution, bronchoscopic sampling, rather than sputum induction, was performed for patients who could not expectorate sputum for the diagnosis of NTM-PD. Although the induction of sputum is considered as one of the sampling methods for patients with suspicion of NTM-PD as well as pulmonary tuberculosis, the superiority of sputum induction over routine sputum examination is questionable [31]. In addition, the optimal methodology for sputum induction has not been established yet [11, 12]. Furthermore, sputum induction was reported to be inferior to bronchoscopy for the diagnosis of pulmonary tuberculosis [32]. Given the decent lung function of our patients in bronchoscopy group, performing bronchoscopy rather than sputum induction to obtain specimen could be regarded as standard care [33].

The strength of this study was that the analyses were based on the prospective cohort, which adopted not only interviews and questionnaires, but objective measures including pulmonary function tests and chest CT scans. Pulmonary function and CT scores taken at baseline evaluation confirmed that both groups were similar in disease severity. Furthermore, disease progression, determined by the on-duty physician during follow-up, was also confirmed by worsening lesions on the CT, which was scored by board-certified radiologists who were blinded to the patients’ clinical data.

In this study, only culture conversion was adopted as the treatment response, which could be a limitation. Because follow-up durations were relatively short after initiating treatment, other treatment outcomes such as ‘microbiological cure’ or ‘treatment failure’ could not be compared for this study. Long-term observation of these patients could elucidate treatment responses more clearly. Another limitation of this study is that rates of both disease progression and culture conversion may have been affected by the different follow-up durations in different groups. Follow-up durations varied according to the time of enrolment.

## Conclusions

In conclusion, disease progression and treatment response were similar between patients with NTM-PD diagnosed from sputum specimens and those diagnosed from bronchoscopic specimens, despite some symptoms and quality of life being worse in patients diagnosed from sputum. We recommend bronchoscopic sampling for patients in whom NTM-PD is suspected clinically or radiographically, especially those who have no or scanty sputum.

## Additional file

Additional file 1: **Table S1.** Laboratory findings for patients enrolled in the nontuberculous pulmonary disease cohort. (DOCX 21 kb)

## Abbreviations

ATS: American Thoracic Society
BTS: British Thoracic Society
CI: Confidence interval
CT: Computed tomography
FEV_1_: Forced expiratory volume in 1 second
FVC: Forced vital capacity
HR: Hazard ratio
IDSA: Infectious Diseases Society of America
IQR: Interquartile range
MAC: *Mycobacterium avium* complex
NTM: Nontuberculous mycobacteria
PD: Pulmonary disease
