# Supplementary material for: Nontuberculous mycobacterial pulmonary disease diagnosed by two methods: a prospective cohort study
Source: BMC Infect Dis. 2019 May 24;19:468. doi: 10.1186/s12879-019-4078-0 (PMC6534935; doi:10.1186/s12879-019-4078-0)
Supplement: Supplementary file 1 — Table S1. Laboratory findings for patients enrolled in the nontuberculous pulmonary disease cohort. (DOCX 21 kb) [file 12879_2019_4078_MOESM1_ESM.docx]

**Table S1 -** Laboratory findings for patients enrolled in the nontuberculous pulmonary disease cohort

| Variables | Total  patients  N=347 | Sputum  group  n=279 | Bronchoscopy group  n=68 | *P-*value |
| --- | --- | --- | --- | --- |
| Serum samples |  |  |  |  |
| White blood cell, x1000/µL | 6.45 (5.24, 7.71) | 6.47 (5.28, 7.77) | 6.29 (5.08, 7.70) | 0.332 |
| Haemoglobin, g/dL | 13.3 (12.4, 13.9) | 13.2 (12.4, 13.9) | 13.4 (12.3, 14.0) | 0.509 |
| Platelet, x1000/µL | 230 (195, 272) | 231 (193, 273) | 228 (198, 264) | 0.773 |
| Blood urea nitrogen, mg/dL | 15 (12, 18) | 15 (12, 18) | 15 (13, 18) | 0.666 |
| Creatinine, mg/dL | 0.79 (0.69, 0.95) | 0.79 (0.69, 0.93) | 0.82 (0.69, 1.02) | 0.328 |
| Total protein, g/dL | 7.4 (7.2, 7.7) | 7.4 (7.2, 7.7) | 7.4 (7.2, 7.6) | 0.882 |
| Albumin, g/dL | 4.3 (4.1, 4.4) | 4.3 (4.1, 4.4) | 4.3 (4.1, 4.5) | 0.151 |
| Total bilirubin, mg/dL | 0.6 (0.5, 0.8) | 0.6 (0.5, 0.8) | 0.6 (0.5, 0.8) | 0.385 |
| C-reactive protein, mg/dL | 0.11 (0.01, 0.39) | 0.11 (0.01, 0.41) | 0.04 (0.01, 0.22) | 0.060 |
| Pulmonary function test results |  |  |  |  |
| FEV_1_, L | 2.15 (1.81, 2.61) | 2.12 (1.79, 2.54) | 2.33 (1.85, 2.80) | 0.060 |
| FVC, L | 2.84 (2.37, 3.44) | 2.83 (2.37, 3.43) | 2.87 (2.33, 3.54) | 0.619 |
| FEV_1_/FVC, % | 77 (71, 82) | 76 (71, 81) | 79 (75, 84) | 0.005 |
| DLCO, % | 93 (83, 105) | 93 (83, 104) | 94 (83, 108) | 0.621 |

Values are presented as median (interquartile range).

FEV_1_, forced expiratory volume in 1 second; FVC, forced vital capacity; DLCO, diffusing capacity of the lungs for carbon monoxide.
